# Long-Term Therapy Outcomes When Treating Chronic Kidney Disease Patients with Paricalcitol in German and Austrian Clinical Practice (TOP Study)

**DOI:** 10.3390/ijms18102057

**Published:** 2017-09-28

**Authors:** Nicholas Obermüller, Alexander R. Rosenkranz, Hans-Walter Müller, Dennis Hidde, András Veres, Sabine Decker-Burgard, Isolde Weisz, Helmut Geiger

**Affiliations:** 1 Division of Nephrology, III, Medical Clinic, University Hospital Frankfurt, Goethe-University, Theodor-Stern-Kai 7, 60590 Frankfurt, Germany; obermueller@em.uni-frankfurt.de; 2 Clinical Division of Nephrology, Department of Internal Medicine, Medical University Graz, Auenbruggerplatz 27, A-8036 Graz, Austria; alexander.rosenkranz@medunigraz.at; 3 AbbVie Deutschland GmbH & Co. KG, Mainzer Strasse 81, 65189 Wiesbaden, Germany; hans-walter.mueller@web.de (H.-W.M.); dennis.hidde@abbvie.com (D.H.); andras.veres@abbvie.com (A.V.); sabine.decker-burgard@abbvie.com (S.D.-B.); 4 AbbVie GmbH, Lemböckgasse 61, A-1230 Wien, Austria; isolde.weisz@abbvie.com

**Keywords:** parathormone (PTH), paricalcitol, secondary hyperparathyroidism (sHPT), chronic kidney disease (CKD), dialysis, predialysis

## Abstract

Paricalcitol is approved for prevention and therapy of secondary hyperparathyroidism (sHPT) in patients with chronic kidney disease (CKD), with only short-term data in clinical routine settings. A 12-month observational study was conducted in Germany and Austria (90 centers, 761 patients) from 2008 to 2013. Laboratory values, demographical, and clinical data were documented in 629 dialysis patients and 119 predialysis patients. In predialysis patients, median intact parathormone (iPTH) was 180.0 pg/mL (*n* = 105) at the start of the study, 115.7 pg/mL (*n* = 105) at last documentation, and 151.8 pg/mL (*n* = 50) at month 12, with 32.4% of the last documented iPTH values in the KDOQI (Kidney Disease Outcomes Quality Initiative) target range. In dialysis patients, median iPTH was 425.5 pg/mL (*n* = 569) at study start, 262.3 pg/mL (*n* = 569) at last documentation, and 266.1 pg/mL (*n* = 318) at month 12, with 36.5% of dialysis patients in the KDOQI target range. Intravenous paricalcitol showed more homogenous iPTH control than oral treatment. Combined analysis of all dialysis patients indicated comparable and stable mean serum calcium and phosphate levels throughout the study. Clinical symptoms, such as itching, bone pain, and fatigue, were improved compared with study entry. The spectrum and frequency of adverse events mirrored the known pattern for patients on dialysis. Paricalcitol is efficacious and has a consistent safety profile in sHPT over 12 months.

## 1. Introduction

Secondary hyperparathyroidism (sHPT) develops during the progression of chronic kidney disease (CKD) and affects morbidity and mortality in these patients [1]. This has already been evaluated in patients with a reduced glomerular filtration rate (GFR) as with patients with CKD stages II and III and aggravating in patients with no or only partial residual function on renal replacement therapy [2]. Vitamin D receptors (VDRs) are targets for pharmacological therapy of sHPT. The vitamin D receptor belongs to the superordinate family of steroid and nuclear hormone receptors [3]. As a nuclear transcription factor, it elicits complex physiological regulation in most tissues and cells following binding of its ligand 1,25-dihydroxyvitamin D. VDR is not only involved in the metabolism of calcium and phosphate, but also crucially implicated in events such cell differentiation, proliferation, apoptosis and different immune responses [3]. Importantly, ligand-bound VDR forms heterodimer with 9-*cis*-retinoid X receptor (RXR) couples to a vitamin d responsive element (VDRE) and with the aid of distinct co-regulatory molecules activating and/or repressing vitamin D target genes [4]. There are two principal functional domains of VDR, a conserved NH_2_-terminal DNA binding domain (DBD) and the more variable COOH-terminal ligand binding domain (LBD). DBD, which is a cysteine-rich zinc finger region, and LBD are connected through a hinge region, probably stabilizing the whole complex [3].

Substitution with active vitamin D compounds might lead to a slowing of sHPT progression [5]. For this purpose, vitamin D receptor activators (VDRAs) were synthesized; however, these agents were nonselective.

In an attempt to identify a VDRA that would be more specific and selective, paricalcitol was developed by chemical modification of the side chain and the A-ring of the molecule, resulting in a selective VDRA [6]. Paricalcitol (19-Nor-1alpha,25-dihydroxyvitamin D2) differs from other VDRA such as calcitriol not only in the side chain structure, but in contrast to other compounds, also in an A-ring configuration. In contrast to previous nonselective VDRAs, paricalcitol yields a reduced intestinal calcium absorption via reduced calbindin induction [7], maintaining the parathyroid hormone (PTH) suppression of VDRAs through a distinct cellular downstream signaling pattern, a finding that is also noted in dialysis patients [8]. Of note, after binding to the vitamin D receptor (VDR), paricalcitol recruits different co-regulators in comparison to other VDRAs, impacting gene expression and regulation differently [9,10]. Wu-Wong et al. have shown that paricalcitol regulates different genes in vascular smooth muscle cells (VSMCs) compared with calcitriol and reduces the expression of several genes that are virtually upregulated in the uremic state [11,12]. In addition, in patients with moderate CKD without diabetes, paricalcitol is able to ameliorate the decline in endothelial function [13]. Those results seem to suggest a specific therapeutic role of paricalcitol that is more distinct, acting at different facets of sHPT. Registration trials have documented efficacy of intravenous [14] and oral [15] paricalcitol in dialysis patients, as well as in non-dialysis patients with CKD stages 3 and 4 [16]. After approval of intravenous paricalcitol in the year 2004 in Germany, the oral compound has been approved for treatment of sHPT in 2008.

Although data from several studies have shown the efficacy of paricalcitol in the treatment of patients with sHPT, most results documented treatment with intravenous (IV) paricalcitol, and had a short follow-up time, with the exception of the study by Lindberg et al. [17]. However, the routine use of paricalcitol (i.e., clinical and laboratory efficiency, safety, patient compliance) in daily practice should be studied more closely to increase knowledge in this scope of application. Other medications related and prescribed for sHPT may influence the effects of paricalcitol; moreover, country-specific issues in the treatment of CKD together with sHPT should be considered in this context.

Observational studies are an indispensable tool to confirm data obtained from randomized controlled trials (RCTs) [18]. To this end, an observational study including patients from German and Austrian dialysis centers was designed to obtain information regarding paricalcitol treatment of sHPT in daily routine for an extended period of time (12 months). Specifically, this study encompasses predialysis and dialysis patients and oral and IV paricalcitol dosing within the dialysis patient group. Of note, patients irrespective of their native/active Vitamin D pretreatment mode, or other medications, were included in the observation period and were thus analyzed.

### 1.1. Patients

The study was conducted in sites specializing in the treatment of CKD and in the offices of community-based specialists/nephrologists. Sites taking part in the study were expected to document data on ≥5 patients. For this study, 761 patients were documented by 90 physicians/clinical study sites throughout Germany (79 sites) and Austria (11 sites). The first patient was treated on 24 March 2008 and the last visit was performed on 11 December 2013. The decision to participate in this study was independent of the prescription of therapies (besides paricalcitol prescription). All adult hemodialysis (HD) and peritoneal dialysis (PD) patients as well as predialysis patients with CKD stages 3 to 5 were eligible for study inclusion. The dose and route of application (paricalcitol capsules or the IV drug formulation) were at the physician’s discretion.

Patients aged >18 years with a prescription for paricalcitol according to the summary of product information (SmPC) and no pretreatment with paricalcitol within the past 6 months were included in the study. It was suggested that patients with an intact PTH (iPTH) value of >1000 pg/mL not be included in the study; however, this suggestion was not mandatory.

The study was approved by the ethical committee of the medical faculty, Johann Wolfgang Goethe-Universität Frankfurt (reference number 332/08, 10 November 2008) and was registered under ClinicalTrials.gov.

### 1.2. Study Design and Aims

The study population exclusively consisted of patients with CKD (predialysis CKD stages 3–5 and dialysis) and diagnosis of secondary hyperparathyroidism. A sample size of 748 patients was available for analysis (in total, 761 patients comprised the safety population).

**Figure 1:**
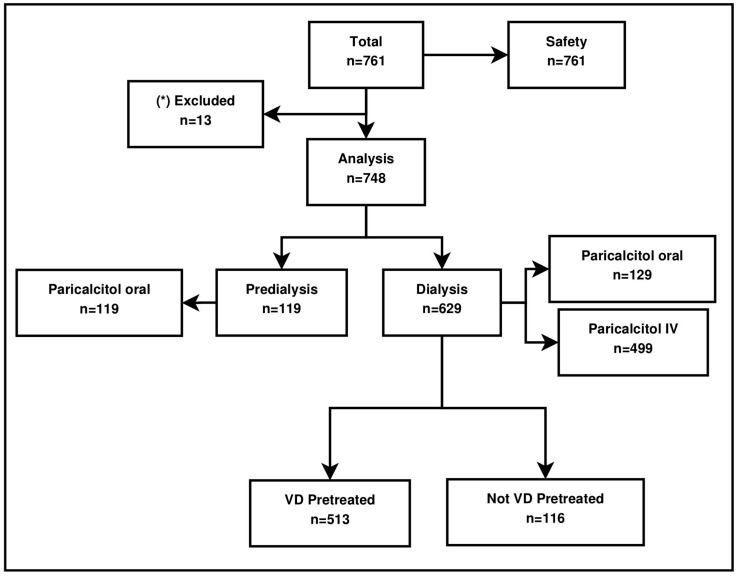
Study population. IV, intravenous; VD, vitamin D. * Insufficient follow-up data available.

Paricalcitol was provided to patients on an on-label basis in an everyday setting. An observation period of 12 months was chosen to observe real-world treatment of patients with oral and IV paricalcitol. The study profile is detailed in Figure 1; the vast majority of the patients received paricalcitol intravenously.

As specific primary study objectives, the proportion of patients attaining an iPTH within the Kidney Disease Outcomes Quality Initiative (KDOQI) [19] target range (CKD stage 3, 35–70 pg/mL; CKD stage 4, 70–110 pg/mL, CKD stage 5, 150–300 pg/mL) in the study period was evaluated, including the time when KDOQI target levels were first achieved and whether these target levels were sustained. However, it should be noted that during the study enrollment, the Kidney Disease Improving Global Outcomes (KDIGO) guidelines [5] were issued, thereby expanding the PTH range (150–300 pg/mL) to “approximately two to nine times the upper normal limit for the assay”. For this study, conventional PTH assays/second-generation immunometric PTH assays were employed. Further laboratory blood parameters assessed included calcium, phosphate, hemoglobin, blood urea nitrogen, C-reactive protein, creatinine, albumin, and alkaline phosphatase.

Secondary objectives of the study were the analysis of episodes with elevated serum calcium and serum phosphate levels in different modality groups. Hypercalcemic values were defined as >2.79 mmol/L. In addition, data regarding the clinical consequences of sHPT (e.g., itching, bone pain, fatigue) were obtained using a visual analog scale (VAS). Furthermore, general safety analyses were conducted.

All data, including safety and compliance specifications, were obtained from case report forms for documentation in Germany; in Austrian sites, electronic data report forms were used. Data were obtained at baseline (month 0), after two weeks, and thereafter monthly up to 12 months for dialysis patients. Data collection from patients on predialysis was done at baseline (month 0), and then quarterly up to month 12. Data concerning adverse events (AEs) and serious adverse events (SAEs) were reported by the participating physicians regardless of type of paricalcitol medication.

### 1.3. Drug

Paricalcitol was administered as soft capsules (1 or 2 µg per capsule), in 1 mL-vials (5 µg) or in 2 mL-vials (10 µg) manufactured by Abbott/AbbVie under the trade name Zemplar^®^.

### 1.4. Statistical Methods

Descriptive statistical analysis was applied using SAS version 9.2 (SAS Institute, Cary, NC, USA) and R version 3.2.2 (The R Foundation, Vienna, Austria). Unavailable values were categorized as either non-evaluable or missing in the case that data were not retrievable or incomplete/fragmentary. For description of medical symptoms, both predialysis and dialysis patient groups were stratified by VAS < 2 and VAS ≥ 2. Statistical tests (Wilcoxon rank sum test, two-sided) were performed to determine the difference between parameters at month 0 and month 12 and were exploratory. All other statistical comparisons and tests were applied exclusively in an exploratory sense.

## 2. Results

### 2.1. Patient Characteristics

Baseline Demographic Data of Study Patients Are Presented in Table 1. Prevalent causes for CKD and for end-stage renal failure, as well as main secondary diagnoses, are listed in Table 2 and Table 3, respectively. Due to more than one simultaneously prevailing, potentially causative diagnosis for CKD, in some cases up to two categories were indicated by physicians. However, nephropathies due to diabetes and arterial hypertension were the leading diagnosis.

The distribution of CKD stages of predialysis patients at study start (determined by eGFR Creatinine equation from the Chronic Kidney Disease Epidemiology Collaboration (CKD-EPI) [20]) and the number of patients in and out of the respective KDOQI iPTH targets are shown in Table 4 (predialysis patients) and Table 5 (dialysis patients).

**Figure 2:**
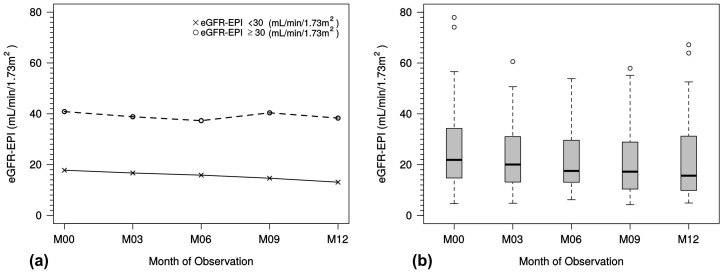
(**a**) Course of eGFR (CKD-EPI) month 0 to month 12 in the predialysis population, stratified by eGFR ≥ 30 and eGFR < 30 mL/min/1.73 m^2^; (**b**) course of eGFR (CKD-EPI) in the predialysis population. Box-whisker-plot shows median and interquartile range with outliers (open circles). eGFR, estimated glomerular filtration rate; M, month.

There was a minimal trend to lower eGFR values during the observation period in patients with an eGFR < 30 mL/min/1.73 m^2^ (Figure 2a), whereas patients with eGFR ≥ 30 mL/min/1.73 m^2^ experienced only minimal changes in eGFR. This is indicated for all predialysis patients in the box-whisker plot analysis (Figure 2b).

Different groups of vitamin D pretreatment modes in predialysis and dialysis patients are shown in Table 6.

The vast majority of patients were pretreated with either a vitamin D compound or combination of vitamin D compounds. A considerable number of patients had no pretreatment; however, this category also included patients with failure of documentation for vitamin D medication. During the observation period, a shift from oral to IV treatment mode in dialysis patients was encountered in 7.9% of cases (10/127). Duration of CKD and sHPT are presented in detail in Table 7 and Table 8.

In the course of the observation, 10 patients in the predialysis group needed dialysis therapy. In 11 patients, referral to kidney transplantation was documented.

The initial dose of paricalcitol treatment, including median dose per week for all patients, is outlined in Table 9. Application mode and concomitant calcimimetic medication at study start are shown in Table 10. Approximately one-third of patients also received cinacalcet.

### 2.2. Medication and Therapy Outcomes

#### 2.2.1. Paricalcitol Titration

**Figure 3:**
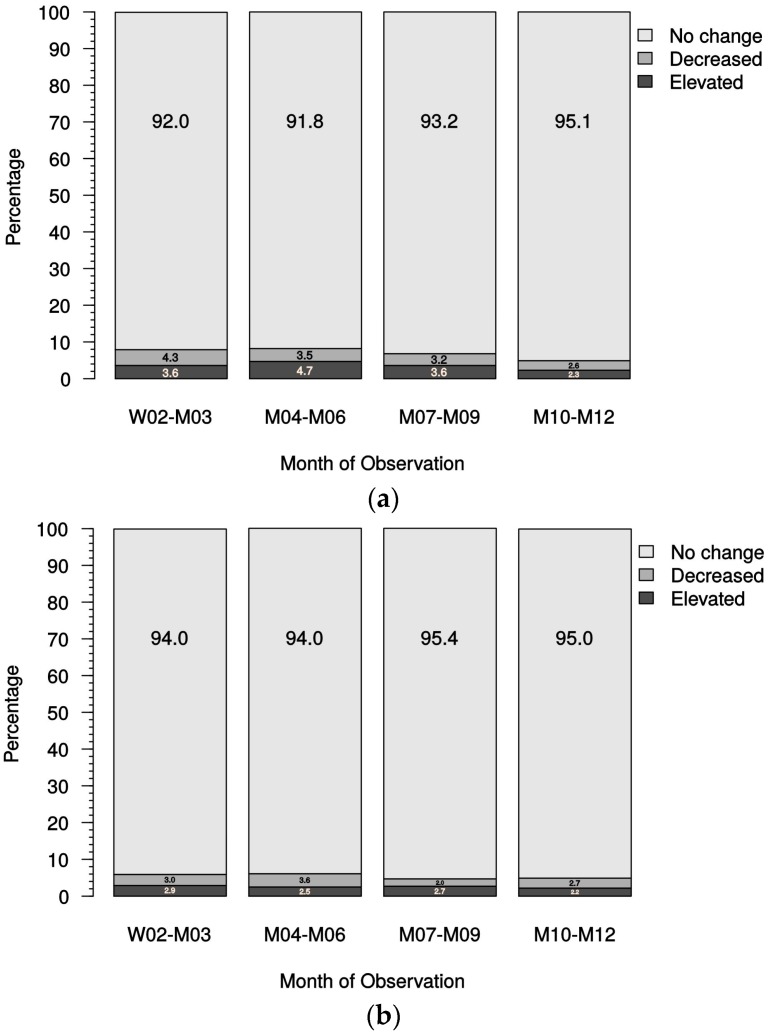
(**a**) Cumulated dose change in patients receiving oral paricalcitol; (**b**) cumulated dose change in patients receiving IV paricalcitol. M, month; W, week.

As outlined in Figure 3a,b overall dose variations were infrequent and generally occurred in the first months, subsequently decreasing during the observation period in both the patients receiving oral and IV paricalcitol. The indication to increase or reduce the weekly dose of paricalcitol was at the discretion of the individual physician; however, the reason for dose variation was not documented.

#### 2.2.2. Phosphate Binder

The spectrum of concomitant phosphate binder prescription is detailed in Table 11.

#### 2.2.3. Laboratory Data

Laboratory values for main blood parameters were documented and are presented for predialysis patients (Table 12) and for dialysis patients stratified for cinacalcet co-medication (Table 13 and Table 14). Medians, number of observations, and minimum to maximum values are presented. 

#### 2.2.4. iPTH Level Trends

**Figure 4:**
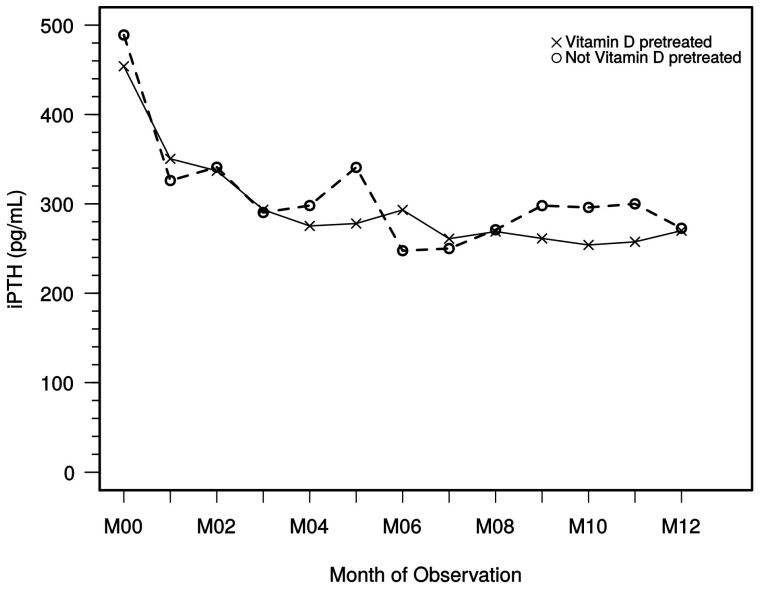
Course of median iPTH in dialysis patients. iPTH, intact parathyroid hormone; M, month; VD, vitamin D.

The overall iPTH values at study start ranged from 24.1 to 2930 pg/mL. All documented iPTH values were included in the analysis. iPTH levels of dialysis patients during study observation demonstrated a fast decline in the first month, followed by an additional drop and stabilization after approximately 4 months (Figure 4). Previous use of native or active vitamin D use and concomitant native vitamin D use (reported in 469 of 629 dialysis patients) did not have a relevant influence on the effect on iPTH trend levels.

The median iPTH values of predialysis patients at study start were 180.0 pg/mL (*n* = 105), the median value of the last documented iPTH was 115.7 pg/mL (*n* = 105), and median iPTH at month 12 was 151.8 pg/mL (*n* = 50).

In the respective predialysis CKD groups, value of iPTH at study start/last iPTH documented/iPTH documented at month 12 were 107.2/82.8/91.1 pg/mL for CKD stage 3 (*n* = 28), 215.4/111.6/128.7 pg/mL for CKD stage 4 (*n* = 46) and 318.4/260.5/311.0 pg/mL for CKD stage 5 (non-dialysis; *n* = 30), respectively.

In the predialysis patient group, 32.4% of last documented iPTH values were in the KDOQI target corridors. In detail, 32.1%, 32.6%, and 33.3% were in the KDOQI target corridors for CKD stage 3, CKD stage 4, and CKD stage 5 (non-dialysis), respectively.

The median iPTH values of dialysis patients at study start were 425.5 pg/mL (*n* = 569), the median value of the last documented iPTH was 262.3 pg/mL (*n* = 569), and the median iPTH at month 12 was 266.1 pg/mL (*n* = 318).

**Figure 5:**
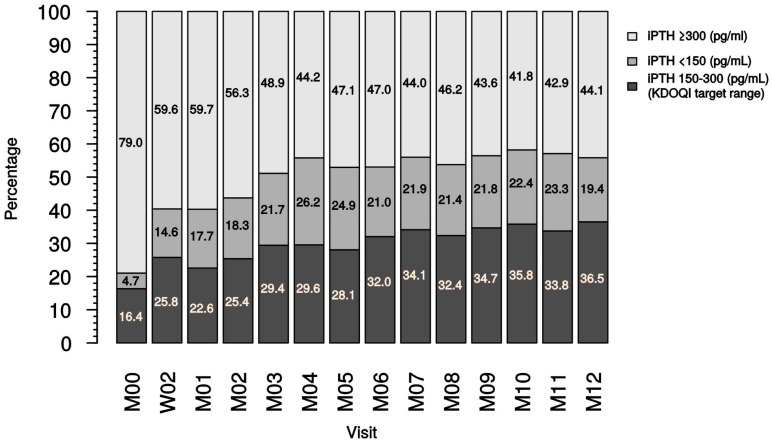
Distribution of iPTH ranges during the duration of the study (dialysis patient iPTH data). iPTH, intact parathyroid hormone; KDOQI, Kidney Disease Outcome Quality Initiatives; M, month; W, week.

In detail, when analyzing different study time points in dialysis patients (the analysis comprised only patients with existing iPTH data), a proportional increase of patients reaching KDOQI target range was noted at month 4, which was then maintained to month 12 (Figure 5). At month 12, 36.5% of dialysis patients and 35.0% of the last documented iPTH values were in the KDOQI target range, displaying a clear increase from month 0 (study start).

**Figure 6:**
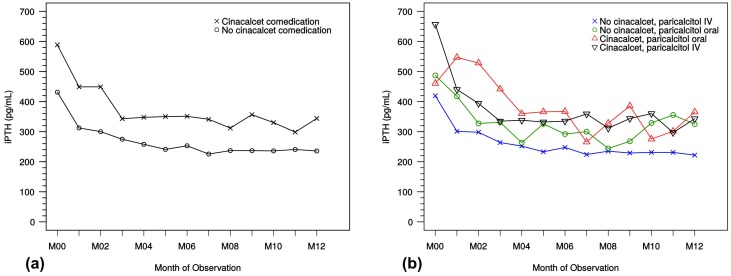
(**a**) Course of iPTH in dialysis patients with and without cinacalcet co-medication; (**b**) course of iPTH in dialysis patients receiving IV and oral paricalcitol with and without cinacalcet co-medication. iPTH, intact parathyroid hormone; M, month. IV, intravenous; M, month.

The course of iPTH values in dialysis patients with or without cinacalcet therapy is shown in Figure 6a. A comparison of iPTH course addressing oral versus IV paricalcitol treatment with regard to concomitant cinacalcet therapy in dialysis patients is shown in Figure 6b. In all shown subgroups, the decrease in median PTH values from month 0 to month 12 was statistically significant (*p* < 0.001, Wilcoxon rank sum test with continuity correction).

Concomitant cinacalcet therapy was defined as a minimum period of ≥90 days. The median number of days with cinacalcet co-medication was 344 days.

For dialysis patients, the median time to KDOQI target was 146 (±98) days. Regarding the subpopulation receiving paricalcitol only (*n* = 365, at month 0), 68.8% of patients (*n* = 260) reached the KDOQI target range within a median time of 148 (±98) days. Of the patients receiving paricalcitol together with cinacalcet (*n* = 198 at month 0), 61.1% (*n* = 121) reached the KDOQI target range within a median time of 141 (±98) days. Thus, the findings were similar in both subpopulations.

#### 2.2.5. Calcium–Phosphate Level Trends

**Figure 7:**
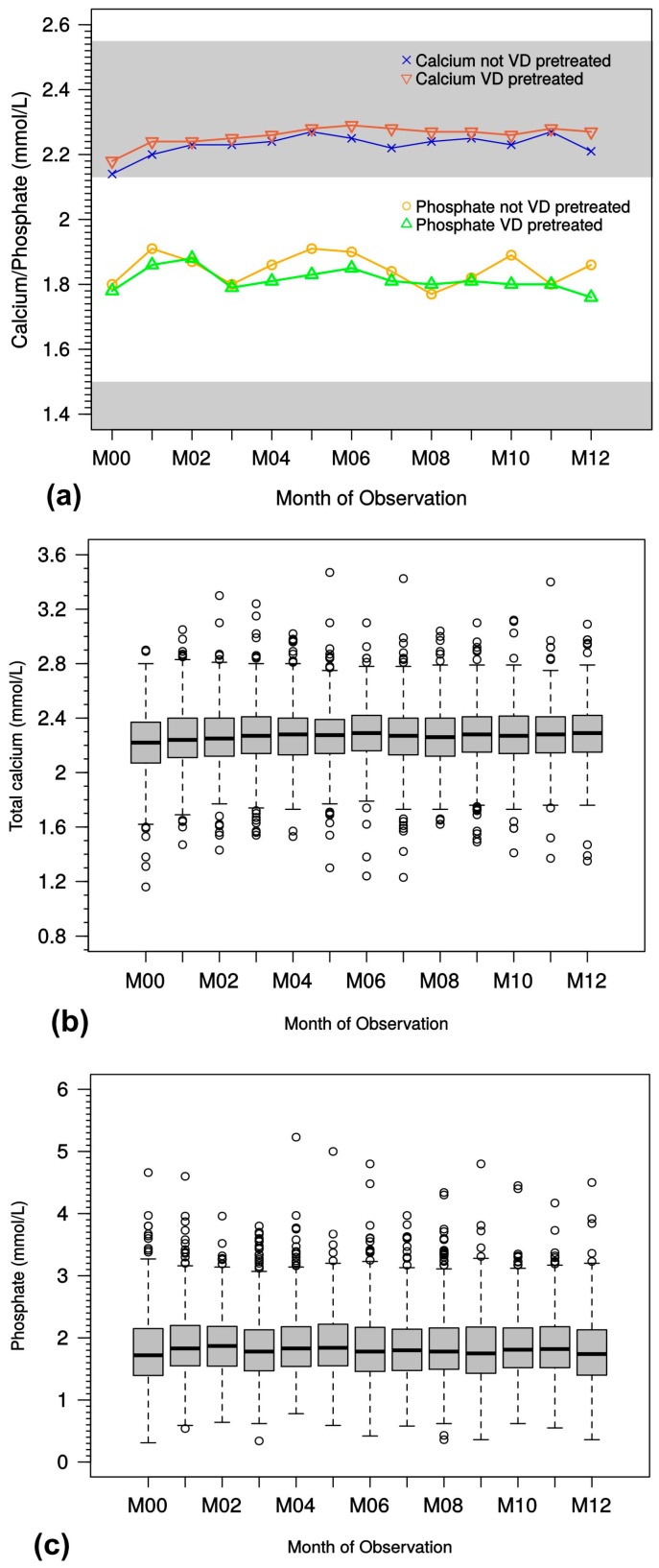
(**a**) Course of median total serum calcium and phosphate levels. Differently colored background areas indicate normal serum levels for either electrolyte. (**b**) Course of total serum calcium levels. Box-whisker-plot shows median and interquartile range with outliers (open circles); (**c**) Course of total serum phosphate levels. Box-whisker plot shows median and interquartile range with outliers (open circles). M, month; VD, vitamin D.

In the overview of all treated patients, including those receiving oral and IV paricalcitol therapy with different modes of vitamin D pretreatment/concomitant treatment (Figure 7a) and detailed by boxplot analysis (Figure 7b,c), stable serum calcium and phosphate levels were observed during the observation period.

**Figure 8:**
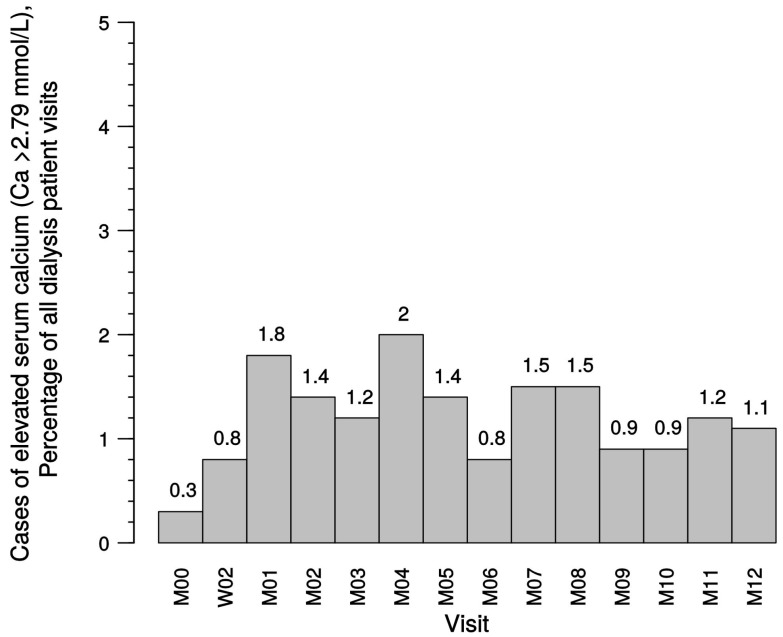
Percentages of elevated serum calcium (>2.79 mmol/L) in dialysis patients. Ca, calcium; M, month; W, week.

In dialysis patients, elevated serum calcium episodes (>2.79 mmol/L) were only encountered rarely and infrequently (in <2% of all visits after study start; see Table 15) and were not found to increase significantly toward the end of the observation period compared with the total course of the study (Figure 8). In predialysis patients, cases of elevated calcium levels were also in the range of 2% of all visits (Table 15).

The slightly higher percentage of elevated calcium values in patients receiving cinacalcet co-medication subjects might reflect advanced sHPT with prior requirement of calcimimetic therapy.

#### 2.2.6. Assessment of Clinical Symptoms Using a VAS

Clinical symptoms were measured using a VAS with a range from 0 to 10 cm. For the specific symptoms “bone pain” and “itching”, the lowest rating was “none” and the highest rating was “severe”. For the symptom “fatigue”, the lowest rating was “little” and the highest rating was “very tired”. The symptoms represented the status of the past two weeks and were documented monthly for dialysis patients and every third month for predialysis patients. Therefore, although a few centers also documented medical symptoms every month in the predialysis group, only the values at month 0, 1, 3, 6, 9, and 12 should be taken into account. Both predialysis and dialysis patient groups were stratified by VAS < 2.0 and VAS ≥ 2.0, with VAS < 2.0 rated as “mild” and VAS ≥ 2.0 as ″moderate to severe″ symptoms. Statistical tests (Wilcoxon, two-sided) were performed for the difference between month 0 and month 12.

**Figure 9:**
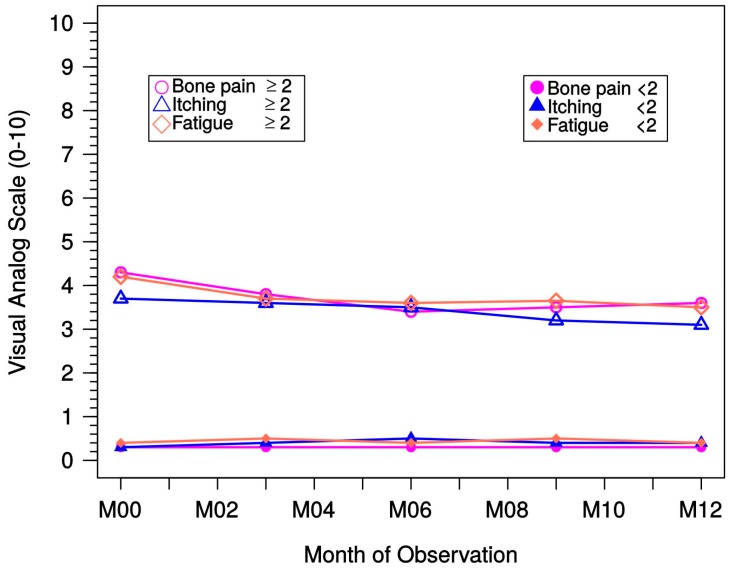
Medical symptoms of dialysis patients stratified by intensity of pain as measured using a VAS. M, month; VAS, visual analog scale.

Both predialysis and dialysis patients with “mild” symptoms experienced no significant changes in symptoms. This was also true for the predialysis group and VAS < 2.0. Respective data from dialysis patients are presented in Figure 9; for better graphical presentation, the 10-cm scale was applied.

In the dialysis patient group and VAS ≥ 2.0, bone pain continuously decreased from 4.6 ± 2.0 to 4.2 ± 1.8 (mean ± SD; *p* = 0.005). In addition, itching scores decreased from 4.2 ± 1.9 to 3.7 ± 1.8 (*p* = 0.001) and fatigue scores decreased from 4.5 ± 1.9 to 4.0 ± 1.8 (*p* = 0.005).

#### 2.2.7. Compliance/Early Termination of Documentation

Compliance was documented by answering the question “Any remarks according to the compliance”. In 14 patients, the physician presumed “noncompliance” regarding the intake of oral paricalcitol medication.

The most prominent reasons (other than death) for discontinuation before month 12 (as noted by discontinuation of documentation) included leaving the dialysis center, kidney transplantation, and others (see Table 16). Of note, discontinuation of documentation did not necessarily mean that patients terminated therapy.

#### 2.2.8. Safety Analysis

Adverse events (AEs), including serious adverse events (SAEs), were generated and coded according to the Medical Dictionary for Regulatory Activities (MedDRA^®^) [21] after reconciliation with AbbVie Pharmacovigilance. In a number of cases, AEs were directly reported to AbbVie Pharmacovigilance without documentation in the case report form. A total number of 728 AEs were documented in 304 patients during the observation period. In Table 17 and Table 18, the MedDRA-coded AEs are separately listed for predialysis and dialysis groups; three documented AEs could not be assigned to one of the groups. Summarizing all available sources, a total of five patients (4.2%) in the predialysis group and 57 patients (9.1%) in the dialysis group died during the observation period.

## 3. Discussion

A disadvantage of only considering RCTs for investigating novel drugs lays in the fact that study populations are not always representative of real-world patient populations. By contrast, in observational studies, the inclusion criteria are often broader and there are wider ranges of coexisting illnesses and disease burden, disease severity, and concomitant treatments. Results from database studies such as the present TOP study can be applied more generally to the entire CKD population and also reflect treatment preferences. The evidence contained within these databases may contribute to clinical decision-making and allows the efficacy and safety of therapy to be assessed. Specifically in nephrology, the important and complementary role of both RCTs and observational studies is appreciated [22].

The present observational study gives insights into the routine management of sHPT in patients with advanced CKD, including dialysis, with a focus on paricalcitol therapy during a 12-month period. Importantly, this study included patients treated with both oral and IV paricalcitol treatment in dialysis patients and oral treatment in predialysis patients, extending data from shorter study periods focusing on treatment with IV paricalcitol [15,23].

Because of the non-interventional nature of this study, no site monitoring was conducted. Because of this, the data quality, including feedback on queries, is not as robust and complete as in an interventional study.

In our study, we also analyzed patients from the predialysis stage (CKD stage 3, CKD stage 4 and CKD stage 5 non-dialysis). Data from the latter group are of high relevance; these patients represent a very inhomogeneous group—with timely unforeseeable deterioration of kidney function with regard to end-stage kidney disease. Some of these patients might rapidly progress to end-stage kidney disease and dialysis, while others will not, making valid interpretation of pharmacotherapeutic effects difficult.

Stable function was observed in patients with an eGFR ≥ 30 mL/min/1.73 m^2^; patients with an eGFR < 30 mL/min/1.73 m² showed a further slight decline within the study period. There was no obvious change in iPTH in either group (data not shown). Whether oral paricalcitol therapy helps stabilize iPTH values in patients in the predialysis phases of CKD cannot be confirmed by this observation.

Recent data from Coyne et al. [24] indicated favorable efficacy in iPTH suppression comparing paricalcitol (−52%) and calcitriol (−46%), with low incidences of hypercalcemia, in patients with stages 3 and 4 CKD. In contrast to our observational study, dosing was mainly based on iPTH, calcium, and phosphorus levels.

The impact of previous and concomitant vitamin D therapy (active/native) on iPTH levels has also been evaluated. Interestingly, our study data did not show relevant differences between the dialysis patients with previous vitamin D therapy and those without. Moreover, no obvious influence of previous vitamin D therapy was observed with regard to calcium and/or phosphate levels during the study period.

The TOP study was conducted at a time when treatment guidelines for sHPT treatment were transitioning from KDOQI in 2003 [19] to KDIGO in 2009 [5]. Since the aforementioned guidelines allowed higher maximum permissible iPTH values, this may have had an influence on the therapy provided to patients, essentially in the prescription practice of paricalcitol, but also of native vitamin D, phosphate binders, and other sHPT-related therapies. It is conceivable that the dosage of paricalcitol and/or native vitamin D might have been tapered in some cases in patients with low iPTH levels, according to KDOQI, had been achieved. However, there was no effect on the overall efficacy in lasting PTH reduction.

Of note, the clinical practice of administering native vitamin D in patients with stage 3 or 4 CKD 3/4 stage and maintaining this treatment in combination with active vitamin D in patients with stage 5 CKD undergoing dialysis is reflected in this study. We did not find an increased risk for hypercalcemia or prolonged hypercalcemia in patients receiving paricalcitol-containing therapy. This is in accordance with the findings of the IMPACT study [25], which also reported only a low incidence of mild hypercalcemia in patients in the paricalcitol treatment arms.

Neither the optimal dosing nor the most beneficial 25-OH vitamin D serum levels are known in patients with CKD, nor are there currently consented repletion strategies. In this regard, KDIGO guidelines from 2009 suggested the therapeutic correction of 25-OH D deficiency and insufficiency for patients with CKD but not on dialysis, but did not provide guidance for dialysis patients [5]. In clinical practice, however, many physicians will tend to supplement vitamin D. The risk of hypercalcemic episodes or values will be dependent on the level of combined (native/active) dosage and the application interval. Here, paricalcitol is offering some biochemical advantages, as shown in a recent clinical investigation pointing to decreased intestinal calcium absorption with paricalcitol compared with calcitriol [8].

Calcimimetics, like cinacalcet, are a class of drugs that suppress PTH secretion by increasing the sensitivity of the parathyroid gland calcium-sensing receptors without increasing plasma calcium levels. The impact of cinacalcet on different parameters has been investigated as well. Although the influence of calcimimetic use in parallel to paricalcitol treatment can only be described with caution, in this study, there were no significant changes in comparison with patients receiving paricalcitol as monotherapy. iPTH, serum calcium, and phosphate levesl and the decrease in PTH levels was similar between the combination therapy and paricalcitol monotherapy groups. This supports the use of cinacalcet in daily routine. The observation that initial median iPTH levels were higher in the group taking cinacalcet (see Table 5 and Table 6) may indicate that it is common practice to prescribe cinacalcet in patients with strongly elevated iPTH levels and possibly refractory disease. Although the initial slope in iPTH decrease was comparable in both groups, the absolute median iPTH values remained on a higher level in patients with cinacalcet. Nevertheless, paricalcitol proved to be effective also in these patients.

Phosphate levels are considered an independent predictor of survival in patients on dialysis and start increasing as eGFR drops below 30 mL/min/1.73 m^2^. Among different factors determining phosphate levels (i.e., residual renal function, dietary intake of phosphate, dialysis adequacy, dose of phosphate binders), the use of vitamin D analogues might play a major role in this scenario.

A recent meta-analysis of CKD in patients who were not undergoing treatment with HD also indicates the efficacy of paricalcitol in lowering PTH levels, but with reference to a careful use of vitamin D analogues because of hypercalcemia and calcification risks [26]. However, in patients with early CKD, including subjects with mild sHPT, those treated even with high doses of native (inactive) cholecalciferol reported no hypercalcemic events [27], indicative of the tolerance of native vitamin D treatment in this group. This is also important for combinations with active vitamin D compounds. In addition, Coyne et al. reported a low incidence of hypercalcemia in patients with stages 3 and 4 CKD treated with paricalcitol while achieving sustained PTH suppression [24].

Although KDIGO guideline recommendations have liberalized maximum permissible values for PTH in sHPT, data from ARO [28] and FARO [29] studies in dialysis patients argue for the achievement of only modestly elevated PTH levels to attain favorable outcomes. This target range is corresponding to the monitoring plan of our observational study according to KDOQI and was reached with paricalcitol in many dialysis patients, but treating physicians may have pursued individual target goals in their patients. Given the sustained reduction as shown by median iPTH levels after 4 or 5 months and in the sequential follow-up (see Figure 4), it does not appear that a marked dose reduction or even cessation of treatment has taken place. Moreover, the data show that in the hands of the treating physicians, paricalcitol dose corrections were not necessary in the majority of patients after several months, indicating an overall safe and sustained treatment effect.

The inability to suppress severe sHPT with the use of traditional VDRA agents can lead to the need for parathyroidectomy, with the patient still at risk for the development of recurrent hyperparathyroidism or aplastic bone disease. In this regard, potent pharmacologic treatment is mandatory, with paricalcitol offering an effective treatment option in the setting of daily clinical practice.

Rapid and significant reduction of iPTH in patients with stage 5 CKD undergoing HD/PD was observed under investigational study conditions [15] within a few weeks. Our observational data support that under real-world conditions, stable results can be obtained within two to three months of treatment and can be maintained for 12 months.

The present observational data add valuable information to phase III clinical trial data investigating the use of paricalcitol in appropriate patients [14,16,17,30] and demonstrated a safety profile that did not reveal any new safety concerns during its use in daily routine practice.

Moreover, compared to historical comparison groups, there was no incidence of increased specific AEs [31]. Regarding the occurrence of deaths, our data do not differ from reported numbers generated from the US DOPPS (Dialysis Outcomes Practice Patterns Study) analysis arm [32], from DOPPS data analysis worldwide [33], or from European DOPPS cohorts [34] or those in Germany [35].

The present observational data regarding PTH suppression are highlighted by the concomitant amelioration of symptoms such as itching, bone pain, and fatigue, which was documented during 12 months of treatment.

Although compliance is generally not a challenge with IV administration, there is a risk of nonadherence to treatment with oral paricalcitol. In 19 patients (7.6%) receiving oral treatment, a total of 33 cases of noncompliance were reported by physicians.

## 4. Conclusions

In conclusion, this long-term observational study underscores the safety and efficacy of IV and oral paricalcitol treatment in predialysis and dialysis patients, providing a sustained decrease in iPTH over a period of 12 months.

## Figures and Tables

**Table 1:** Demographic data.

Parameter	Predialysis				Dialysis			
	*n*	Mean	SD	Range	*n*	Mean	SD	Range
Age, years	119	64.4	13.9	31–86	629	62.5	14.9	23–90
Height, cm	119	170.0	8.9	150–192	626	169.5	9.6	142–207
Weight, kg	119	85.7	18.4	46–132.8	628	78.7	19.4	33.2–178
Male	73				345			
Female	46				284			
Duration of CKD, months	117	72.2	99.8	0–482	613	79.2	73.1	0–509
Duration of sHPT, months	94	19.5	23.7	0–142	396	45.6	38.3	0–249
Peritoneal dialysis, *n*					23			
Hemodialysis, *n*					603			

CKD, chronic kidney disease; SD, standard deviation; sHPT, secondary hyperparathyroidism; *n*, number of patients.

**Table 2:** Causes of chronic kidney disease.

Cause	Frequency	Percentage
Diabetic nephropathy	223	29.4
Glomerulonephritis	181	23.9
Hypertensive nephropathy	150	19.8
Polycystic kidney disease	67	8.8
Pyelonephritis	31	4.1
Interstitial nephritis	24	3.2
Other	144	19.0
Unknown	102	13.4

**Table 3:** Secondary diagnoses.

Diagnosis	Frequency	Percentage
Coronary heart disease	246	32.40
Heart failure	138	18.20
Myocardial infarction	64	8.40
Obesity	180	23.70
Arterial hypertension	645	85.00
COPD	43	5.70
Type 2 diabetes mellitus	276	36.40
Anemia	498	65.60
Renal osteodystrophy	67	8.80
Dyslipidemia	248	32.70
Other	381	50.20

COPD, chronic obstructive pulmonary disease.

**Table 4:** Predialysis population at study start: CKD Stages and Attainment of iPTH Targets.

CKD Stage	eGFR Range, mL/min/1.73 m^2^	Number of Patients in CKD Stage	Number of Available iPTH Data at Study Start	iPTH KDOQI Target Range, pg/mL	iPTH within KDOQI Target Range, *n* (%)
2	60–89	2	1	35–70	1 (100.0%)
3	30–59	33	28	35–70	5 (17.9%)
4	15–29	51	46	70–110	8 (17.4%)
5 (non-dialysis)	<15	30	30	150–300	8 (26.7%)
eGFR n/a		3			
Total		119	105		22 (21.0%)

CKD, chronic kidney disease; eGFR, estimated glomerular filtration rate; iPTH, intact parathyroid hormone; KDOQI, kidney disease outcomes quality initiatives.

**Table 5:** Dialysis population at study start: iPTH KDOQI target attainment.

iPTH (pg/mL)	No Co-Medication		Cinacalcet Co-Medication	
	*n*	Percentage	*n*	Percentage
150–300 (KDOQI target range)	93	14.79	1	0.61
<150	27	4.29	17	10.43
≥300	454	72.18	118	72.39
Data not available	55	8.74	27	16.56
Dialysis total	629	100	163	100

iPTH, intact parathyroid hormone; KDOQI, kidney disease outcomes quality initiatives; *n*, number of patiens.

**Table 6:** Vitamin D pretreatment for the predialysis and dialysis population.

Population	Drug	Category	Total Population	Treated Patients	Percentage
Predialysis	Calcitriol, alfacalcidol	Active VD	119	44	36.97
	ergocalciferol, cholecalciferol	Native VD	119	35	29.41
	Combination	Active and native VD	119	8	6.72
	No VD pretreatment		119	31	26.05
	Data not available		119	1	0.84
Total			119	119	100
Dialysis	Calcitriol, alfacalcidol	Active VD	629	208	33.07
	ergocalciferol, cholecalciferol	Native VD	629	119	18.62
	Combination	Active and native VD	629	179	28.46
	No VD pretreatment		629	115	18.28
	Data not available		629	8	1.27
Total			629	629	100

VD, vitamin D.

**Table 7:** Duration of CKD in months before study start.

Population	*n*	Min	Q25	Mean	SD	Median	Q75	Max
Analysis total	742	0.0	28.7	79.0	78.8	57.2	99.5	508.7
Predialysis	118	0.0	16.3	71.8	99.4	44.2	80.0	482.0
Dialysis	624	0.1	30.9	80.3	74.3	59.4	103.2	508.7
No VD pretreatment	148	0.1	26.2	75.6	73.1	59.5	101.2	433.3
Active VD pretreatment	435	0.6	31.2	83.8	83.4	58.90	100.7	508.7
Native VD pretreatment	334	0.0	27.9	74.8	74.3	54.0	87.80	508.7
Cinacalcet pretreatment	163	2.0	45.9	100.7	83.2	78.5	126.9	428.1
Peritoneal dialysis	22	9.7	34.6	107.7	108.7	50.7	173.6	320.9
Hemodialysis	600	0.1	30.4	79.0	72.6	59.4	101.1	508.7

Max, maximum; Min, minimum; Q, quartile; SD, standard deviation; VD, vitamin D; *n*, number of patients.

**Table 8:** Duration of sHPT in months before study start.

Population	*n*	Min	Q25	Mean	SD	Median	Q75	Max
Analysis total	498	0.00	12.90	40.40	37.10	31.20	58.00	248.70
Predialysis	95	0.00	2.60	19.40	23.60	11.00	31.40	142.10
Dialysis	403	0.00	18.60	45.40	38.00	35.30	66.50	248.70
No VD pretreatment	91	0.00	0.90	26.50	39.40	9.00	32.50	231.00
Active VD pretreatment	282	0.00	19.50	45.50	35.40	36.50	63.70	248.70
Native VD pretreatment	230	0.00	18.30	43.10	35.60	34.50	60.30	248.70
Cinacalcet pretreatment	91	0.10	35.20	64.90	41.00	61.30	87.10	231.00
Peritoneal dialysis	15	2.50	13.80	49.60	47.70	40.50	74.00	190.70
Hemodialysis	389	0.00	18.30	45.00	37.70	35.10	65.00	248.70

Max, maximum; Min, minimum; Q, quartile; SD, standard deviation; VD, vitamin D; *n*, number of patients.

**Table 9:** Weekly dose (mcg) of paricalcitol: statistical characteristics at study start.

Group	*n*	Min	Q25	Mean	SD	Median	Q75	Max
Predialysis, paricalcitol oral	118	1.00	5.00	6.50	2.80	7.00	7.00	18.00
Dialysis, paricalcitol oral	127	1.00	6.00	7.20	4.00	6.00	7.00	28.00
Dialysis, paricalcitol IV	497	2.50	5.00	11.70	7.10	10.00	15.00	70.00

IV, intravenous; Max, maximum; Min, minimum; Q, quartile; SD, standard deviation; *n*, number of patients.

**Table 10:** Route of application at study start in the dialysis population.

Route of Application	Dialysis (*n* = 629)	Percentage of Dialysis Patients	Cinacalcet Co-Medication, *n*	Cinacalcet Co-Medication, % of Subgroup
Paricalcitol oral	129	20.5	68	52.7
Paricalcitol IV	499	79.3	161	32.3
Data n/a	1	0.2		

IV, intravenous; n/a, not available; *n*, number of patients.

**Table 11:** Summary of phosphate binders at study start.

Type of Phosphate Binder	Number of Prescriptions	Percentage of All Prescriptions	Predialysis Prescriptions	%	Dialysis Prescriptions	%
Aluminum-containing	168	21.8	3	18.8	165	21.9
Calcium-containing	239	31.0	6	37.5	233	30.9
Lanthan	133	17.2	0	0	133	17.6
Sevelamer	227	29.4	6	37.5	220	29.1
Unknown type	4	0.5	1	6.3	3	0.4
Total	772	100	16	100	755	100

**Table 12:** Relevant laboratory data from predialysis patients *.

Laboratory Parameter	Month 0	Month 6	Month 12
Albumin, g/L	49 (39; 18–49)	41 (37; 20–66)	42 (29; 28–65)
Alkaline phosphatase, U/L	89.0 (83; 30.0–208.8)	88.0 (57; 25.0–308.4)	74.5 (42; 23.0–244.8)
Blood urea nitrogen, mg/dL	55.8 (70; 15.6–175.0)	65.8 (50; 12.4–136.0)	75.2 (42; 14.5–162.3)
Serum calcium, total, mmol/L	2.3 (108; 1.8–2.9)	2.4 (79; 1.9–2.8)	2.4 (63; 2.0–2.7)
Serum calcium, ionized, mmol/L	1.2 (11; 1.0–2.2)	1.1 (9; 0.8–1.3)	1.2 (8; 1.0–1.4)
Serum creatinine, µmol/L	2.5 (109; 1.1–9.8)	3.1 (79; 1.0–9.7)	3.2 (61; 0.9–11.5)
C-reactive protein, mg/L	3.0 (82; 0.2–53.0)	4.3 (57; 0.4–296.0)	2.5 (45; 0.4–204.0)
eGFR-EPI, mL/min/1.73 m^2^	22.4 (109; 4.7–78.0)	17.7 (79; 6.2–53.8)	16.6 (61; 4.9–92.2)
Hemoglobin, g/dL	12.2 (109; 7.6–17.1)	12.3 (79; 9.2–17.8)	12.3 (63; 9.1–17.2)
Serum phosphate, mmol/L	1.2 (108; 0.3–3.6)	1.3 (78; 0.4–4.5)	1.4 (60; 0.4–2.8)
25-OH-vitamin D, µg/L	18.0 (75; 3.5–85.4)	22.7 (32; 2.6–42.8)	23.7 (30; 2.0–56.8)

eGFR, estimated glomerular filtration rate. * Data are presented as medians (*n*; min–max).

**Table 13:** Relevant laboratory data from dialysis patients *.

Laboratory Parameter	Month 0	Month 6	Month 12
Albumin, g/L	40 (323; 20–65)	40 (212; 22–58)	39 (202; 23–48)
Alkaline phosphatase, U/L	95.0 (311; 36.0–483.0)	79.2 (240; 24.0–267.0)	81.0 (220; 26.4–477.0)
Blood urea nitrogen, mg/dL	80.9 (299; 2.2–391.4)	78.0 (243; 3.7–411.4)	77.0 (230; 3.1–287.5)
Serum calcium, total, mmol/L	2.2 (378; 1.5–2.9)	2.3 (303; 1.2–3.1)	2.3 (278; 1.4–3.1)
Serum calcium, ionized, mmol/L	1.1 (117; 0.5–2.7)	1.1 (115; 0.6–2.2)	1.1 (89; 0.6–1.4)
Serum creatinine, µmol/L	7.5 (385; 1.1–15.2)	8.1 (301; 1.8–16.9)	8.3 (277; 1.6–15.8)
C-reactive protein, mg/L	5.1 (331; 0.0–500.0)	5.0 (266; 0.0–122.0)	5.0 (246; 0.0–137.0)
Hemoglobin, g/dL	11.6 (394; 7.7–17.0)	11.7 (334; 7.8–15.6)	11.6 (298; 7.0–18.3)
Serum phosphate, mmol/L	1.8 (391; 0.6–4.7)	1.8 (312; 0.4–4.8)	1.8 (284; 0.4–3.8)
1,25-(OH)_2_-vitamin D, pg/mL	26.4 (59; 1.5–160.8)	26.2 (34; 1.3–109.0)	12 (38; 1.3–67.2)
25-OH-vitamin D, µg/L	21.6 (199; 2.0–81.8)	24.0 (123; 0.3–76.5)	22.0 (118; 2.5–79.6)

* Data are presented as medians (*n*; min–max).

**Table 14:** Relevant laboratory data from dialysis patients with cinacalcet co-medication *.

Laboratory Parameter	Month 0	Month 6	Month 12
Albumin, g/L	40.0 (168.0; 23.0–50.0)	39.5 (114; 24.0–49.0)	40.0 (107; 23.0–64.0)
Alkaline phosphatase, U/L	103.4 (188; 39.0–417.0)	94.0 (145; 37.6–486.6)	93.0 (123; 42.0–365.0)
Blood urea nitrogen, mg/dL	99.5 (182; 28.1–245.0)	93.0 (146; 2.0–322.9)	83.1 (125; 9.0–222.0)
Serum calcium, total, mmol/L	2.2 (220; 1.3–2.7)	2.3 (174; 1.4–2.9)	2.3 (158; 1.4–3.0)
Serum calcium, ionized, mmol/L	1.1 (29; 0.6–1.3)	1.1 (30; 0.7–1.4)	1.1 (23; 0.5–1.3)
Serum creatinine, µmol/L	8.3 (226; 1.0–16.3)	8.8 (176; 0.7–16.4)	8.5 (159; 2.3–16.7)
C-reactive protein, mg/L	4.1 (183; 0.0–120.0)	5.0 (139; 0.0–317.0)	5.0 (130; 0.0–311.0)
eGFR-EPI, mL/min/1.73 m^2^	n/a	n/a	n/a
Hemoglobin, g/dL	11.7 (227; 7.7–14.8)	11.8 (190; 8.5–17.0)	11.8 (163; 8.4–15.7)
Serum phosphate, mmol/L	1.8 (226; 0.9–3.8)	1.9 (178; 0.8–3.6)	1.8 (160; 0.7–4.5)
1,25-(OH)_2_-vitamin D, pg/mL	20.2 (19; 0.5–60.0)	11.7 (14; 3.2–45.6)	12.0 (13; 0.5–33.6)
25-OH-vitamin D, µg/L	22.9 (78; 4.0–60.5)	24.4 (52; 2.0–69.0)	24.0 (47; 2.0–68.6)

eGFR, estimated glomerular filtration rate. * Data are presented as medians (*n*; min–max). n/a, not applicable.

**Table 15:** Events of elevated serum calcium (>2.79 mmol/L) after study start.

Patient Group	Number of Visits after Study Start	Documented Events of Elevated Serum Calcium, *n*	%
Dialysis patients (w/cinacalcet)	2160	40	1.85
Dialysis patients (paricalcitol only)	3845	38	0.99
Dialysis patients (all)	6005	78	1.30
Predialysis patients	533	9	1.69

**Table 16:** Reason for early termination of documentation (all patients).

Reason	Frequency
Left dialysis center	29
Other reasons	17
Kidney transplantation	11
Elevated calcium/phosphate	9
Patient’s decision	9
Adverse event	8
Noncompliance	8
Hospitalization	7
PTx	4

**Table 17:** Overview of AEs occurring in predialysis patients.

MedDRA SOC	Frequency	%
Cardiac disorders	10	10.31
Infections and infestations	10	10.31
Metabolism and nutrition disorders	10	10.31
Renal and urinary disorders	10	10.31
Surgical and medical procedures	10	10.31
Gastrointestinal disorders	9	9.28
General disorders and administration site conditions	7	7.22
Nervous system disorders	7	7.22
Musculoskeletal and connective tissue disorders	5	5.15
Respiratory, thoracic and mediastinal disorders	5	5.15
Investigations	4	4.12
Blood and lymphatic system disorders	3	3.09
Injury, poisoning and procedural complications	3	3.09
Skin and subcutaneous tissue disorders	2	2.06
Hepatobiliary disorders	1	1.03
Neoplasms benign, malignant and unspecified (incl. cysts and polyps)	1	1.03

MedDRA, Medical Dictionary for Regulatory Activities; SOC, system organ class.

**Table 18:** Overview of AEs occurring in dialysis patients.

MedDRA SOC	Frequency	%
Surgical and medical procedures	96	15.26
Infections and infestations	87	13.83
Injury, poisoning and procedural complications	68	10.81
Vascular disorders	54	8.59
General disorders and administration site conditions	51	8.11
Gastrointestinal disorders	48	7.63
Investigations	44	7.00
Cardiac disorders	36	5.72
Metabolism and nutrition disorders	24	3.82
Nervous system disorders	23	3.66
Musculoskeletal and connective tissue disorders	20	3.18
Skin and subcutaneous tissue disorders	16	2.54
Neoplasms benign, malignant and unspecified (incl. cysts and polyps)	14	2.23
Respiratory, thoracic and mediastinal disorders	13	2.07
Eye disorders	8	1.27
Reproductive system and breast disorders	6	0.95
Blood and lymphatic system disorders	5	0.79
Psychiatric disorders	4	0.64
Hepatobiliary disorders	3	0.48
Immune system disorders	3	0.48
Congenital, familial and genetic disorders	2	0.32
Endocrine disorders	2	0.32
Ear and labyrinth disorders	1	0.16

MedDRA, Medical Dictionary for Regulatory Activities; SOC, system organ class.
